# Commentary on Mathie RT et al. Method for appraising model validity of randomised controlled trials of homeopathic treatment: multi-rater concordance study

**DOI:** 10.1186/1472-6882-12-240

**Published:** 2012-11-29

**Authors:** Mikel Aickin

**Affiliations:** 1 National College of Natural Medicine, Portland, OR, USA

## Abstract

Although many researchers agree that applying conventional drug-trial quality criteria to CAM studies lacks plausibility, few take on the burden of devising alternative criteria in a specific area of CAM. This commentary points out strengths and weaknesses in the approach taken in the work of Mathie and colleagues to do this for homeopathy.

## Background

In the search for a universal methodology for therapeutic research, the medical research community has invested nearly all of its trust in randomized clinical trials (RCT). To some investigators, however, trying to find the single “best” method for comparing treatments is as misguided as the equally conventional quest for the “best” treatment for all patients with a given condition. These investigators argue that in the same way that medical care directs attention to the individual patient, clinical science should place its focus on the nature of the treatment under study [1,2]. From this standpoint RCTs have developed in a symbiotic fashion with research on drugs, and are admirably suited to that purpose, but may have rather severe problems when they are over-extended into non-drug research.

## Discussion

The article under discussion here [3] approaches this problem by seeking to create and define domains that can be used for assessing the degree to which a homeopathy RCT reflects homeopathic practice. This follows the track laid down in evidence-based medicine by applying various “quality criteria” for rating published studies. Whereas this conventional approach attempts to use criteria that are flexible enough to apply to any RCT, the Mathie et al. article specifically takes on the task of providing criteria that are tailored to homeopathic research studies. Both approaches suffer from their dependence on how the RCTs were described in publication, as opposed to how they were carried out in the real world.

In conventional medicine, the rise of quality criteria (which now exist in dozens of versions; [4]) was in response to problems encountered in writing systematic reviews. It was perceived to be very difficult to summarize the results from multiple publications when there was so much variability in how the trials were described and how the results were presented. Thus projects like CONSORT were oriented toward formalizing how certain kinds of information should be communicated, to make it easier to compile them into systematic reviews [5].

It did not take very long, however, for reporting criteria to be interpreted as scientific criteria. “Reporting quality standards” very quickly became “quality standards” [6]. As such, most lists of quality standards contain no substantive evaluation of the scientific quality of the articles to which they are applied. For example, when it comes to statistical analysis of the results, the only quality scales that even mention this aspect contain some vague comment about “appropriateness”, without further specification. (The one exception to this that I have seen was a very specific list, half of which I regard as mistaken.) The issue this raises is: if it is so difficult to get consensus on what criteria determine the difference between good and not-so-good science publications, then how are we to have any confidence that we are educating researchers to be able to tell the difference?

From this viewpoint Mathie et al. can be seen as an attempting to say in the simplest possible terms, what distinguishes genuine homeopathy research from something that only seems like homeopathy research. I would imagine there are several reasons why this is a good idea. First, a number of researches into homeopathy have been designed, funded, and carried out by individuals who had insufficient understanding of how homeopathy is actually practiced. Thus at least three of Mathiie et al.’s six domains depend on expert homeopathic judgment, in one way or another. Secondly, when funding for homeopathy research became available relatively recently, the pool of potential homeopathy-savvy investigators was generally weak in medical research knowledge or experience, leading to the publication of studies that could be impugned on scientific grounds. Thus, the other three of Mathie et al.’s criteria ask for scientific judgments about the appropriateness of the research design (again relying to some extent on an understanding of homeopathy).

Given that the objective of Mathie et al. is worthy, I think one can question whether their work is extensive enough, since it involved only six evaluated articles by eight reviewers. In addition, evidently the reviewers are the same ones who were involved in the consensus development of the criteria, whereas it is generally more realistic to test judgment-based criteria with a fresh set of reviewers. More evidence is needed on the degree of reviewer concordance, and in a more representative pool of articles.

It was of some interest to me that Mathie et al. did not endorse the combination of their six items into a scale. For some time now it has seemed to me that adding item scores requires some considerable justification, which may actually be absent. For example, if the investigators did not blind subjective outcome assessments, they should get a zero on that item, and it should then be *multiplied* by the sum of the other items. In other words, a critical error should not have the effect of merely diminishing an overall quality score; it should obliterate the score.

## Conclusions

It seems fairly clear to some readers of systematic reviews that in their attempts to be “objective” in applying their subjective standards, reviewers may have achieved a level of uniformity in the format for their judgments that actually reduces their intrinsic validity. I have been impressed over the years, in preparing discussions of research articles for classroom use, how many different ways there are for researchers to introduce poor practices into their studies, most of which are not captured by any of the many conventional quality scales. Nevertheless, one must recognize that having rating systems with no rules amounts to going back to the bad old system we had before the era of evidence-based medicine, and so attempts like that of Mathie et al. should be praised and encouraged for trying to bring the review of homeopathy RCTs into the world of relevant clinical science.

## Competing interests

The author has no competing interests to declare.

## Authors’ contributions

MA drafted and revised the manuscript.

## Pre-publication history

The pre-publication history for this paper can be accessed here:

http://www.biomedcentral.com/1472-6882/12/240/prepub

## References

[B1] Heron J Critique of conventional research methodology Complement Med Res 1986 1 1 12 22

[B2] Cartwright N Are RCTs the Gold Standard? BioSocieties 2007 2 11 20 10.1017/S1745855207005029

[B3] Mathie RT Roniger H Van Wassenhoven M Frye J Jacobs J Oberbaum M Bordet M-F Nayak C Chaufferin G Ives JA Dantas F Fisher P Method for appraising model validity of randomized controlled trials of homeopathic treatment: multi-rater concordance study BMC Med Res Methodol 2012 12 49 10.1186/1471-2288-12-49 22510227 PMC3394086

[B4] Olivo SA Macedo LG Gadotte IC Fuentes J Stanton T Magee DJ Scales to assess the quality of randomized controlled trials: a systematic review Phys Ther 2008 88 2 156 175 10.2522/ptj.20070147 18073267

[B5] Begg C Cho M Eastwood S Horton R Moher D Olkin I Pitkin R Drummon R Schulz D Stroup D Improving the quality of reporting randomized controlled trials: the CONSORT statement JAMA 1996 276 8 637 639 10.1001/jama.1996.03540080059030 8773637

[B6] Hammerschlag R Milley R Colbert A Weih J Yohalem-Ilsley B Mist S Aickin M Randomized controlled trials of acupuncture (1997–2007): an assessment of reporting quality with a CONSORT- and STRICTA-based instrument Evid-Based Compl Alt 2011 2011 25 Article ID 183910 10.1155/2011/183910 PMC2952291 20953418

